# Novel Throat-Attached Piezoelectric Sensors Based on Adam-Optimized Deep Belief Networks

**DOI:** 10.3390/mi16080841

**Published:** 2025-07-22

**Authors:** Ben Wang, Hua Xia, Yang Feng, Bingkun Zhang, Haoda Yu, Xulehan Yu, Keyong Hu

**Affiliations:** 1 School of Information Science and Engineering (SISE), Hangzhou Normal University, Hangzhou 311121, China; 20170056@hznu.edu.cn (B.W.); xiahua@stu.hznu.edu.cn (H.X.); zhangbingkun08@stu.hznu.edu.cn (B.Z.); yuhaoda0205@stu.hznu.edu.cn (H.Y.); 2024210214052@stu.hznu.edu.cn (X.Y.); hukeyong@hznu.edu.cn (K.H.); 2 Mobile Health Management System Engineering Research Center of the Ministry of Education, Hangzhou 311121, China; 3 Zhejiang-Cyprus Smart City and Mobile Health Joint Laboratory, Hangzhou 311121, China

**Keywords:** throat-attached sensors, PVDF, deep belief network, Adam optimizer

## Abstract

This paper proposes an Adam-optimized Deep Belief Networks (Adam-DBNs) denoising method for throat-attached piezoelectric signals. The method aims to process mechanical vibration signals captured through polyvinylidene fluoride (PVDF) sensors attached to the throat region, which are typically contaminated by environmental noise and physiological noise. First, the short-time Fourier transform (STFT) is utilized to convert the original signals into the time–frequency domain. Subsequently, the masked time–frequency representation is reconstructed into the time domain through a diagonal average-based inverse STFT. To address complex nonlinear noise structures, a Deep Belief Network is further adopted to extract features and reconstruct clean signals, where the Adam optimization algorithm ensures the efficient convergence and stability of the training process. Compared with traditional Convolutional Neural Networks (CNNs), Adam-DBNs significantly improve waveform similarity by 6.77% and reduce the local noise energy residue by 0.099696. These results demonstrate that the Adam-DBNs method exhibits substantial advantages in signal reconstruction fidelity and residual noise suppression, providing an efficient and robust solution for throat-attached piezoelectric sensor signal enhancement tasks.

## 1. Introduction

Speech recognition technology is essential for modern applications, including virtual assistants, automatic transcription services, and human–computer interaction systems. However, background sounds can significantly interfere with the clarity of speech signals in noisy environments, resulting in a reduced recognition accuracy. The limitations of conventional acoustic microphones have prompted researchers to turn to alternatives. Among these alternatives, laryngeal microphones (i.e., larynx-attached piezoelectric transducers) have attracted attention due to their unique advantages. By directly capturing vibration signals from a speaker’s larynx, laryngeal microphones can effectively isolate ambient noise and enable reliable speech processing in complex conditions [1].

Various methods have been proposed by researchers to enhance the performance of laryngeal microphones in speech recognition. Spectral mapping techniques are used to convert signals from throat microphones into a form more suitable for conventional identification systems [2]. The Gaussian Mixture Model (GMM) for learning is utilized to enhance the spectral envelope and excitation signals of speech [3]. The EGC (Equalization Generation Combined) Framework has significant advantages in the field of laryngeal voice enhancement [4]. While numerous methods have been proposed to optimize throat microphones, they continue to encounter various challenges. These approaches often require substantial quantities of labeled data for training and tend to have limited generalization capabilities across different noise environments. Additionally, some techniques rely on the integration of throat microphones with conventional microphone signals; however, this combination is not always practical in real-world applications.

Electrical signals frequently exhibit complex nonlinear noise couplings, which present significant challenges in acquiring large amounts of labeled data—an undertaking that is both expensive and time-consuming. This scenario highlights the pressing need for an unsupervised training algorithm capable of effectively extracting features suited for nonlinear modeling. Deep Belief Networks (DBNs) excel in this regard, utilizing a greedy algorithm that facilitates unsupervised pre-training layer by layer. This innovative approach not only adeptly decouples the intricate nonlinear relationships between noise and signal but also thoroughly learns the underlying distribution of the data. Furthermore, DBNs uniquely enable the generation of new data samples based on the insights gained from the trained model, making them an invaluable tool in data analysis [5]. DBNs exhibit a multilayer architecture, which presents a significant challenge in terms of the training process, rendering it time-intensive [6]. In this paper, we present a method for denoising laryngeal piezoelectric signals using Adam-optimized DBNs [7]. This method employs the short-time Fourier transform (STFT) to convert the original signal into the time–frequency domain and the Adam optimization algorithm to enhance the training process of the DBN model, thereby improving its denoising performance through the adaptive adjustment of the learning rate [8]. The initial noise reduction spectrum is subsequently converted back to the time domain and input into the DBN model, which is optimized using the Adam algorithm to achieve complete signal reconstruction. This method effectively utilizes the a priori structure of the noise while also leveraging the depth model’s capacity to capture nonlinear structures.

## 2. Principles of Relevant Algorithms

### 2.1. Data Acquisition

Polyvinylidene fluoride (PVDF) piezoelectric films are commonly used in pneumatic devices [9]. PVDF material exhibits flexibility, a low mass, and a strong mechanical compatibility, making it suitable for high-sensitivity acoustic–mechanical signal conversion [10]. When a PVDF film is subjected to external forces, the film’s surface deforms under this pressure. As a result, the positive and negative charge centers within the material shift due to the inherent asymmetry of the PVDF piezoelectric film’s crystalline structure. This displacement leads to the polarization of the film’s surface, with different regions exhibiting opposite charges. The density of the generated charge is directly proportional to the amount of pressure applied to the piezoelectric film, which is known as the “piezoelectric effect” [11]. According to this piezoelectric effect, the charge generated when the PVDF piezoelectric film receives pressure is related to the external force as follows:

(1) Q=d·F

where Q is the charge output by the film; d is the piezoelectric coefficient, determined by the material properties of the PVDF piezoelectric film itself; and F is the external force applied to the piezoelectric film.

To verify the sign properties of the piezoelectric constants of the PVDF film, one end of the sensor is fixed, while a pressure of 0.0001 N is applied to the other end from the bottom to the top, followed by an application of −0.0001 N. The parameters of the PVDF piezoelectric film used in this simulation are shown as Table 1.

**Figure 1:**
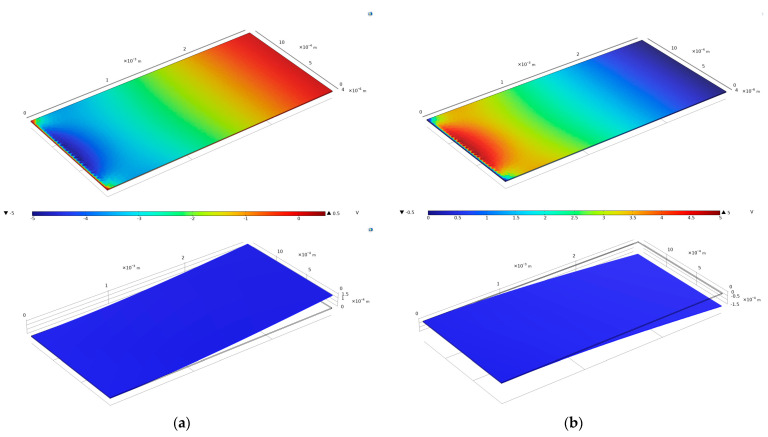
(**a**) Static potential (V) and deformation diagrams of PVDF sensor unit at 0.0001 N pressure. (**b**) Static potential (V) and deformation diagrams of PVDF sensor unit at −0.0001 N pressure.

The map of the simulated surface potential is displayed in Figure 1.

Simulations were conducted to empirically demonstrate the directional sensitivity and charge generation reliability of PVDF film sensors for laryngeal applications by applying forces in different directions. Any small vibration will cause the values collected by the sensors to change accordingly, and this property is well characterized for translating the bi-directional mechanical vibrations of the vocal folds into directional electrical signals for laryngeal vibration sensor applications.

In this paper, a piezoelectric thin-film sensor made of PVDF material was attached to the surface of the larynx of an experimental subject to collect mechanical vibration signals. The sensors were securely affixed to the left and right sides of the thyroid cartilage using transparent adhesive. This setup aimed to ensure stable sensing of the mechanical vibrations generated by the laryngeal muscles and the trachea during natural phonation. All the data were recorded by the same experimenter in a controlled environment to maintain consistency and comparability.

### 2.2. Data Preprocessing

To enhance the time series modeling capabilities and improve the effectiveness of frequency domain noise separation in the subsequent modeling stage, this paper introduces three essential preprocessing operations during the original signal processing stage: trajectory matrix construction, normalization, and short-time Fourier transform. The K nearest reference points are selected, and the unknown location is estimated based on their coordinates.

Given that the original piezoelectric vibration signal is a one-dimensional non-stationary time series, directly processing it effectively to capture its local dynamic characteristics is tricky [12]. For this reason, this paper adopts the sliding window approach to construct the trajectory matrix of the original signal to strengthen its time series structure. Let the original signal be $x=\left[x_{1},x_{2},\ldots ,x_{L}\right]$, the trajectory matrix is constructed in the following way: set the window length to be L, then the resulting trajectory matrix $X\in R^{L\left(T-L+1\right)}$ is constructed with the following expression:

(2) $$X=\left[\begin{array}{ccc} \begin{array}{cc} x_{1} & x_{2}\\ x_{2} & x_{3} \end{array} & \ldots & \begin{array}{c} x_{T-L+1}\\ x_{T-L+2} \end{array}\\ \vdots & \ddots & \vdots \\ \begin{array}{cc} x_{L} & x_{L+1} \end{array} & \ldots & x_{T} \end{array}\right]$$

Each column in the trajectory matrix represents a segment of the signal within a localized time window that has significant temporal context properties.

The simultaneous recovery of the trajectory matrix to the original signal requires the diagonal averaging method, using the trajectory matrix *X* as described in Equation (2); the total number of antidiagonals of the trajectory matrix is D=L+K−1; for the t-th element $\hat{x}$ in the output sequence (where t=(1,2,3….D)), it consists of the average of all elements on the *t*-th diagonal of the trajectory matrix.

(3) $$\hat{x_{t}}=\frac{1}{n_{t}}\sum_{\left(i,j\right)\in \Omega_{t}}x_{i,j}$$

where $\Omega_{t}=\left(i,j\right)$, which are all the elements of the trajectory matrix that satisfy i + j = t + 1. $n_{t}$ is the number of elements on the diagonal t, calculated as follows.

(4) $$n_{t}=\left\{\begin{array}{ll} t & if\;1\leq t\leq min(L,K)\\ min(L,K) & if\;min(L,K)<t\leq max(L,K)\\ L+K-t & if\;max(L,k)<t\leq L+K-1 \end{array}\right.$$

To prevent the amplitude differences between segments from interfering with the spectrum analysis after constructing the trajectory matrix [13], this paper further normalizes the trajectory matrix by columns. Let the j-th column of the trajectory matrix be $x^{j}$, whose mean and standard deviation are $\mu^{j}$ and ${\;\sigma }^{j}$, respectively, then the normalized column vector is calculated as follows.

(5) $${\tilde{x}}^{j}=\frac{x^{j}-\mu^{j}}{\sigma^{j}}$$

During the normalization process, all segments are modeled and analyzed using the same scale. This approach enhances the stability of spectrum extraction. Once normalization is complete, we can further analyze the joint distribution of the signal in both time and frequency. This paper uses the STFT; the expression for STFT is as follows.

(6) $$X\left[m,k\right]=\sum_{n=0}^{N-1}x\left[n+mH\right]\cdot w\left[n\right]\cdot e^{-j2\pi kn/N}$$

where $W\left[n\right]$ is the window function, $x\left[n\right]$ is the normalized signal, m is the time frame index, H is the window step, k is the frequency index, and N is the number of points in the FFT.

When the Fourier transform is applied to a signal that is not strictly periodic, spectral leakage occurs, resulting in distortion in the spectrum. To mitigate this issue, the Hanning window is used. This window smooths the transition to zero at both ends of the signal, helping to reduce spectral leakage due to its characteristic of converging to zero at both ends [14]. In this paper, we utilize the Hanning window, and the expression used is as follows.

(7) $$w[n]=\frac{1}{2}-\frac{1}{2}cos[\frac{2\pi n}{N-1}]$$

The Hanning window can minimize spectral leakage and improve spectral resolution.

To complete the mapping from the frequency domain to the time domain, the signals in the frequency domain need to be transformed back into time domain signals using the inverse STFT method. The expression for inverse STFT is as follows.

(8) $$x\left[n\right]=\frac{1}{N}\sum_{m=-\infty }^{\infty }\sum_{k=0}^{N-1}X\left[m,k\right]\cdot e^{j2\pi kn/N}\cdot w\left[n-mH\right]$$

Currently, the preprocessing module completes the entire closed-loop process, which includes time-domain acquisition, frequency-domain transformation, and signal restoration. This step provides the initial conditions necessary for the subsequent signal reconstruction using a deep neural network in an end-to-end manner.

### 2.3. Deep Denoising Model Based on Adam-DBNs

After completing the frequency-domain feature extraction and the initial suppression of the noise band, residual noise interference and distortion in the time structure still persist. As a result, achieving high-fidelity reconstruction using traditional frequency-domain filtering methods is challenging. To enhance the recovery accuracy of time-domain signals, this paper presents a multilayer nonlinear modeling strategy based on a DBN, combined with the Adam optimization algorithm, to effectively perform the deep denoising of complex signals.

The DBN is a generative deep model made up of multiple stacked Restricted Boltzmann Machines (RBMs). It possesses strong nonlinear modeling capabilities and is well-suited for representing the non-Gaussian and non-smooth distributions of electrical signals [6]. Its basic unit, the Restricted Boltzmann Machine (RBM), is a two-layer undirected graphical model comprising a visible layer, denoted as v, and a hidden layer, denoted as h. The energy function associated with this model is defined as

(9) $$E\left(v,\;h\right)\;=\;-\sum_{i=1}^{D}a_{i}\;v_{i}\;\;-\;\sum_{j=1}^{F}\;b_{j}\;h_{j}\;-\;\sum_{i=1}^{D}\sum_{j=1}^{F}v_{i}\;W_{ij}\;h_{j}\;$$

where $W\in R^{D\times FW}\;$ represents the connection weights, while $a_{i}$ and $b_{j}$ are the bias parameters. For continuous input signals, a Gauss–Bernoulli RBM is used [15], and its energy function can be expressed as follows: the probability distribution in the RBM is determined by the energy function.

(10) $$E\left(v,h\right)=\sum_{i=1}^{D}\frac{{\left(v_{i}-a_{i}\right)}^{2}}{2\sigma_{i}^{2}}-\sum_{j=1}^{F}b_{j}h_{j}-\sum_{i=1}^{D}\sum_{j=1}^{F}\frac{v_{i}}{\sigma_{i}}W_{ij}h_{j}$$

**Figure 2:**
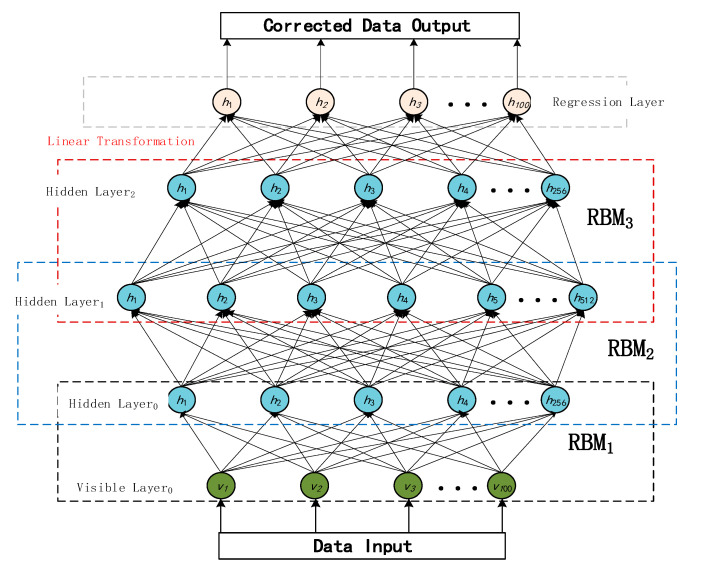
The topology of the DBN used in this paper.

The network is first pre-trained in an unsupervised manner using a layer-by-layer contrast approach. After completing the feature extraction, it is then connected to the regression output layer for fine-tuning. Figure 2 shows the topology of the DBN used for this experiment, which employs a combination of hierarchical unsupervised pre-training and end-to-end supervised fine-tuning.

At the beginning of training, the input signal passes through three RBM layers (RBM_1_ → RBM_2_ → RBM_3_) from the visible layer in order from the bottom up, and the hidden layer of each RBM is transformed with a feature nonlinear transformation using a sigmoid activation function. After the pre-training is completed, in the fine-tuning stage, the output of the top hidden layer is mapped to the regression layer through the linear transformation module, and the final output is the reconstructed signal with the same dimension as the input.

To enhance the model’s convergence speed and training stability, the Adam optimizer is employed for parameter updates. Its main principle combines first-order momentum with second-order momentum estimation [16]. The updated rules are as follows:

(11) $$\left\{\begin{array}{c} m_{t}=\beta_{1}m_{t-1}+\left(1-\beta_{1}\right)\nabla L_{t}\\ v_{t}=\beta_{2}v_{t-1}+\left(1-\beta_{2}\right){\left(\nabla L_{t}\right)}^{2}\\ \hat{m_{t}}=\frac{m_{t}}{1-\beta_{1}^{t}}\\ \hat{v_{t}}=\frac{v_{t}}{1-\beta_{2}^{t}}\\ \theta_{t+1}=\theta_{t}-\alpha \cdot \frac{\hat{m_{t}}}{\sqrt{\hat{v_{t}}}+\epsilon } \end{array}\right.$$

where $\nabla L_{t}$ is the current gradient, α is the learning rate, and $\beta_{1}$ and $\;\beta_{2}$ are the momentum coefficients.

To account for both the reconstruction quality of the waveform structure and spectral features, the joint loss function is designed as follows:

(12) $$L=\lambda_{1}\cdot MSE|x,\hat{x}|+\lambda_{2}\cdot ||X\left(f\right)|-|\hat{X}\left(f\right)|{|}_{1}$$

where $|X\left(f\right)|\;$ and $\left|\hat{X}\left(f\right)\right|$ are the spectral amplitudes of the original and reconstructed signals, respectively, $\lambda_{1}$ = 0.7, $\lambda_{2}$ = 0.3.

This chapter outlines the theoretical basis for the STFT-based frequency-domain noise suppression method and the Adam optimization of DBNs. These concepts establish the groundwork for the implementation of the algorithms that will follow. The next chapter will concentrate on the practical application of these methods, detailing the specific processes for data preprocessing, algorithm design, model training, and the analysis of results from experimental validation.

## 3. Experimental Design and Analysis of Results

To address the challenges of poor signal denoising and the difficulty of maintaining signal integrity in complex noise environments, this study introduces a signal denoising method that combines STFT and DBNs. By attaching PVDF films to both sides of the glottis, this method effectively captures the vibration signals produced during speech. This approach significantly reduces interference from ambient noise and enhances the clarity of the signals.

**Figure 3:**
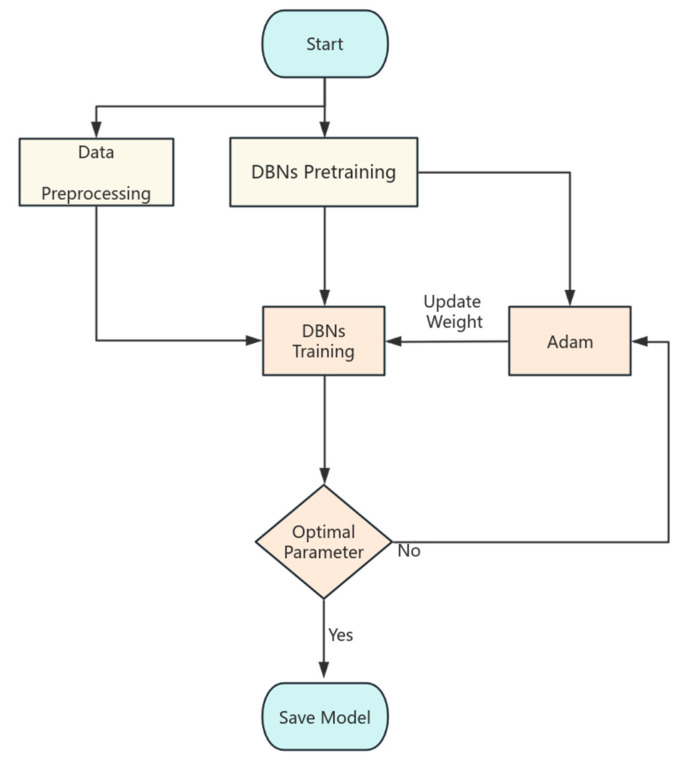
Model training process.

The training dataset consists of English storytelling examples, encompassing various speech patterns and intonation variations. This chapter provides a detailed explanation of the algorithmic implementation of the proposed method, covering four key components: data acquisition and preprocessing, noise suppression techniques, model training and optimization, and signal reconstruction and validation. Figure 3 shows the overall flow of the model training.

**Figure 4:**
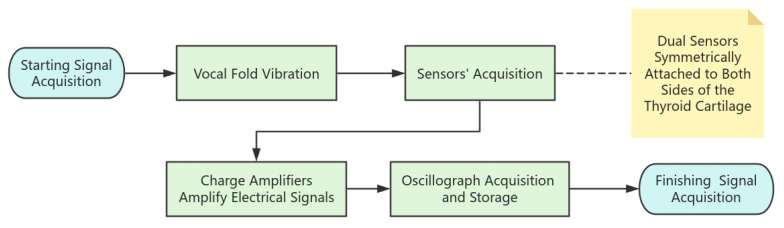
Signal acquisition system flowchart.

**Figure 5:**
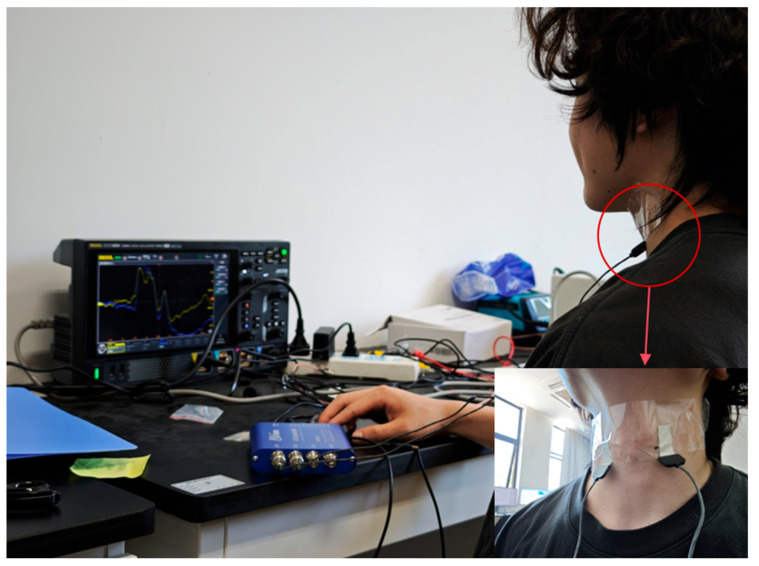
The piezoelectric film is attached to the larynx.

There has been some research progress on the optimal mounting location of throat vibration sensors [7]. In this paper, dual sensors are symmetrically attached to both sides of the thyroid cartilage to directly capture the bi-directional stress generated by vocal fold vibration and realize the non-invasive monitoring of laryngeal muscle movement. The signal acquisition idea for this experiment is shown in Figure 4. As shown in Figure 5, the electrical signals generated when reading the English short story “Standing on the Roof of a Small Goat and the Wolf” are captured. The blue device shown in the figure is a charge amplifier, which amplifies the electrical signals captured during the vibration of the vocal cords.

The tiny vibration signals are converted into weak electrical signals by PVDF sensors, and then the signals are amplified by charge amplifiers and then acquired and stored by oscilloscopes, which is the signal acquisition process, and after the acquisition is completed, the data preprocessing can begin.

**Figure 6:**
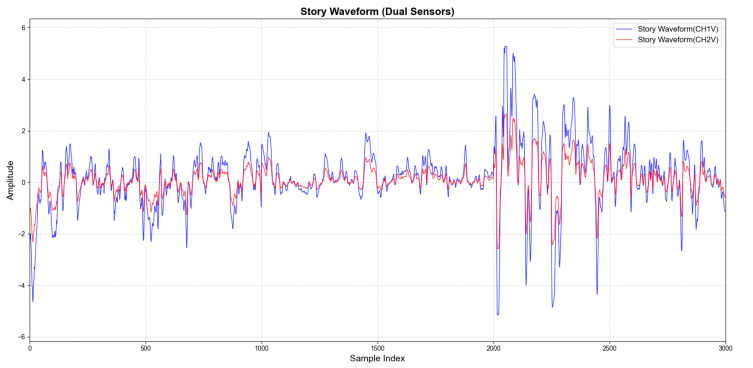
English story waveform.

Figure 6 displays the waveforms of laryngeal tissue vibrations collected using PVDF piezoelectric film sensors positioned on both the left and right sides of the laryngeal node. The changes in amplitude of these waveforms directly reflect the vibration state of the vocal folds. Although the amplitudes of the two waveforms differ, their shapes are essentially similar. This similarity is explained by the principle of superposition in linear systems:

(13) $$y_{total\left(t\right)}=y_{CH1\left(t\right)}+y_{CH2\left(t\right)}$$

After fusing the sensor data in a linear superposition, preprocessing ensues (Equation (2)).

When the waveform displays a pronounced upward trend and remains at a high amplitude level, it indicates a vocal phase in which the vocal folds are actively vibrating. This phase corresponds to the core of voiced consonants and vowels. Conversely, when the waveform rapidly decreases and stays in the low-amplitude region, it signifies that the vocal folds have stopped vibrating. This condition corresponds to the sustained fricative segments of clear consonants and voiceless stops. The steep rising and falling edges of the waveform typically mark the critical moments of Voice Onset and Voice Offset. As illustrated in the figure, the amplitude significantly increases after the point at 2000, which aligns with the experimentally designed vocalization task of tone exacerbation. During tone exacerbation, the amplitude of vibration is greater due to increased tension in the vocal fold muscles. This tension causes the thyroarytenoid muscle to contract, leading to a stiffening of the vocal folds.

The DBN model begins with a pre-training phase, during which the Adam optimizer is not used. In the following formal training process, Adam is introduced to fine-tune and optimize the weights of the DBNs. Once training is complete and the model is saved, the signals that need processing undergo a preprocessing phase. This phase involves creating and normalizing the trajectory matrices. After the preprocessing is finished, the prediction results are transformed back into the original signals using a method called diagonal averaging. This diagonal averaging is crucial for accurately reconstructing the signal.

To quantitatively assess the performance of the denoising model based on DBNs, we have established several evaluation criteria that encompass both time-domain and frequency-domain characteristics [17]. This approach ensures a comprehensive evaluation of the denoising effect. In the frequency domain, spectral flatness is employed to measure the flatness of the noise:

(14) $$\mathrm{Spectral}\;\mathrm{Flatness}=\frac{\exp\left(\frac{1}{N}\sum_{n=1}^{N}\log\left(P_{k}\right)\right)}{\frac{1}{N}\sum_{n=1}^{N}\left(P_{K}\right)}$$

where $P_{k}$ is the power spectral density of the kth frequency component, N is the number of frequency points, the SF value domain is [0, 1], and lower values indicate less noise residuals.

The Pearson product–moment correlation coefficient (PPMCC) was used to measure the similarity between the original signal and the noise reduction signal in the time domain, with the expression

(15) $$\rho_{xy}=\frac{\sum \left(x_{i}-\bar{x}\right)\left(y_{i}-\bar{y}\right)}{\sqrt{\sum {\left(x_{i}-\bar{x}\right)}^{2}\sum {\left(y_{i}-\bar{y}\right)}^{2}}}$$

where $x_{i}$ is the original signal, $y_{i}$ is the noise reduction signal, and $\bar{x}\;\mathrm{and}\;\bar{y}$ is the signal mean. The value range of $\rho_{xy}$ is [−1, 1]; higher means higher similarity.

In this paper, local noise energy (LNE) is also introduced as one of the evaluation metrics to measure the degree of local perturbation of residual noise in the signal after noise reduction. Different from the global metrics, LNE focuses on the local energy fluctuation in each signal frame, which is especially suitable for evaluating the noise suppression ability of the model under a fine-grained time window. By dividing the signal $X\left[n\right]$ into M segments, and when the length of each segment is L,

(16) $$Energy=\frac{1}{M}\sum_{m}^{M}\left(\frac{1}{L}\sum_{n}^{L}(x_{m}[n]-{{\bar{x}}_{m})}^{2}\right)$$

LNE is essentially the “localized intra-frame variance average”, which is used to reflect high-frequency jitter or noise energy.

Combine the three to obtain a new evaluation criterion:

(17) $$Score=100\cdot \left(W_{1}\cdot \rho_{xy}+W_{2}\cdot \left(1-min\left(1,\frac{Flatness}{0.1}\right)\right)+W_{3}\cdot \left(1-min\left(1,\frac{Energy}{0.1}\right)\right)\right)$$

($W_{1}$,${\;W}_{2}$,${\;W}_{3}$) are the weights, set to $\left(0.4,\;0.3,\;0.3\right)$, respectively.

The DBN model used in the experiment was enhanced through 100 rounds of supervised fine-tuning. Figure 6 displays the loss curves for both training and validation losses. It is evident that the Adam optimizer significantly accelerates the convergence process and offers a clearer basis for evaluating the model’s performance.

**Figure 7:**
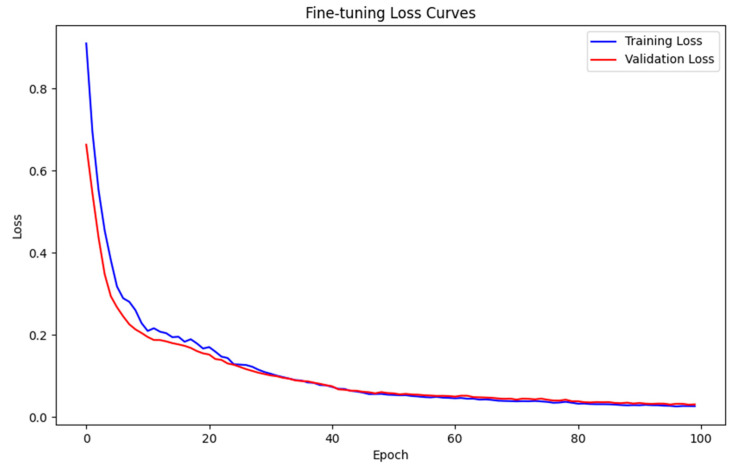
Training and validation loss curves.

Figure 7 shows that the training loss and validation loss decrease rapidly in the first 20 epochs from the initial value of 0.8 to about 0.15, which reflects the efficiency of Adam’s optimizer, and then the curves stabilize, the training loss stabilizes around 0.028, and the validation loss is a little higher but stays at about 0.0305. This suggests that the model fits well with the training data, and no overfitting phenomenon occurs. In order to further quantify the convergence of the model in the fine-tuning stage, Table 2 lists some of the training and validation loss functions of the Adam-DBNs during 100 rounds of training, and it can be seen that the overall training process has a good stability and convergence.

**Figure 8:**
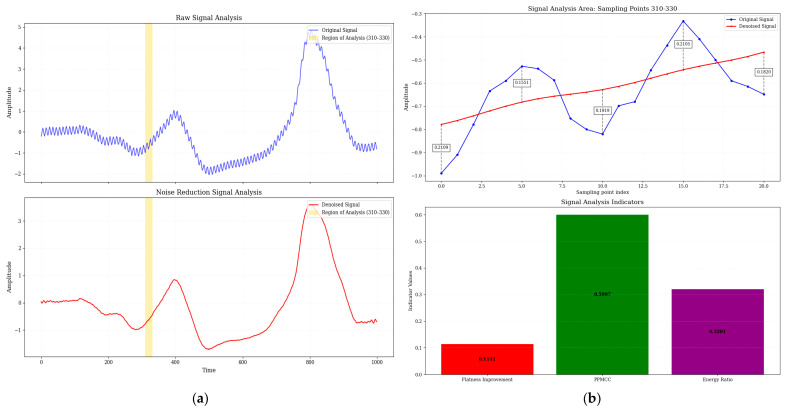
The part of the waveform with the best noise reduction for the word “math” (highlighted by the yellow rectangle in the figure). (**a**) Comparison of original waveform and noise reduction waveform. (**b**) Key waveform areas and detailed metrics (yellow region in (**a**)).

**Figure 9:**
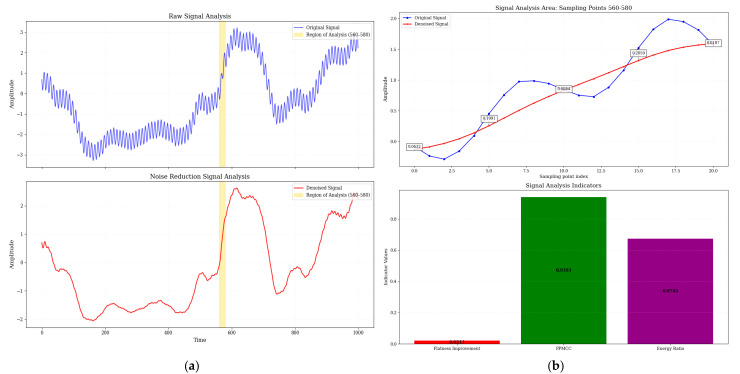
The part of the waveform with the best noise reduction for the phrase “HZNU” (highlighted by the yellow rectangle in the figure). (**a**) Comparison of original waveform and noise reduction waveform. (**b**) Key waveform areas and detailed metrics (yellow region in (**a**)).

Figure 8 and Figure 9 show a comparison of electrical signals before and after denoising by Adam-DBNs. The original signal (Figure 8a and Figure 9a) displays obvious high-frequency perturbations and low-frequency background fluctuations, characterized by unclear waveform edges and distorted periodic structures. After denoising, the irregular oscillations are effectively smoothed while preserving the main characteristics. Figure 8b provides a detailed analysis of the critical region (310–330 sampling points, range of 560–580 in Figure 9b), with the top panel showing the raw and denoised signals and the bottom panel presenting three quantitative metrics: spectral flatness improvement, PPMCC, and energy ratio (ratio of noise reduction signal energy to original signal energy). These results collectively demonstrate the efficacy of Adam-DBNs in enhancing signal quality.

After verifying the noise reduction ability of the proposed Adam-DBNs method in typical samples, this paper further compares it with CNN, which is a common one-dimensional sequence modeling method that has been widely used in speech enhancement and biosignal denoising tasks. In order to make a fair comparison, the comparison model adopts the same input structure and training data as the Adam-DBNs, and the number of training rounds and optimizer settings are also the same. The specific CNN parameters are displayed in Table 3. Table 4 shows the performance comparison of the two methods in terms of several metrics.

From Table 4 and Figure 8, it can be seen that the Adam-DBNs achieve smoothing curves in waveform noise reduction. At the same time, they are closer to the original signals after noise reduction. Their comprehensive scores are significantly higher than those of the CNN models, which further verifies the stability and generalization ability of this paper’s method under noisy conditions.

## 4. Conclusions

In this study, an electrical signal denoising method combining STFT and DBNs is proposed for processing the glottal node vibration signals collected by piezoelectric thin-film sensors in complex noise environments. The method firstly constructs the time–frequency spectrogram by STFT; subsequently, the nonlinear features of the signal are learned by the DBN model, the time-domain reconstruction is completed, and the diagonal averaging method is used in the reconstruction stage to maintain the structural integrity of the temporal sequence.

Existing methods mentioned in the introduction for specific problems, while effective, are often limited by linear or shallow modeling assumptions that make it difficult to thoroughly deal with complex nonlinear noise residuals in the laryngeal signal. The success of this work’s strategy of initial STFT suppression combined with the deep reconstruction of DBNs is keyed to the generative nature and hierarchical unsupervised pre-training of DBNs. Rather than simply learning a mapping function, DBNs efficiently model the intrinsic probability distribution of a clean laryngeal vibration signal through their RBM stacks. This allows the model to not only suppress noise but also “generate” or “reconstruct” a signal structure that better matches the physiological vibration characteristics based on the learned data distribution.

Quantitatively, the proposed Adam-DBNs method achieved a 6.77% improvement in waveform similarity (PPMCC), reduced the local noise energy (LNE) by 0.099696, and improved spectral flatness from 0.001395 to 0.001301, resulting in a composite score increase from 69.07 to 93.33 compared with a baseline CNN. These metrics demonstrate the effectiveness of our model in enhancing the signal quality and reducing noise across both time and frequency domains.

In future work, several promising directions can be explored. First of all, expanding the model’s robustness to more diverse noise conditions and speech patterns, including tonal languages and emotional speech, would improve its generalization. In addition, integrating adaptive windowing strategies in STFT preprocessing may further optimize the resolution trade-off in time–frequency representation. Ultimately, extending the current model into a real-time embedded system could facilitate on-device applications such as voice-controlled wearable systems or silent-speech interfaces. Future research could also investigate hybrid architectures combining DBNs with attention mechanisms or graph-based models to better capture temporal dependencies in biosignals.

## Figures and Tables

**Table 1:** PVDF piezoelectric film parameters.

Parameters	Value
Relative Permittivity	{7.4, 9.3, 7.6}
Elasticity Matrix Pa	$\left(\begin{array}{c} \begin{array}{cccccc} 3.8\times 10^{9} & 1.9\times 10^{9} & 0.9\times 10^{9} & 0 & 0 & 0\\ 0 & 3.8\times 10^{9} & 0.9\times 10^{9} & 0 & 0 & 0\\ 0 & 0 & 1.2\times 10^{9} & 0 & 0 & 0\\ 0 & 0 & 0 & 9\times 10^{8} & 0 & 0\\ 0 & 0 & 0 & 0 & 9\times 10^{8} & 0\\ 0 & 0 & 0 & 0 & 0 & 7\times 10^{8} \end{array} \end{array}\right)$
$\mathrm{Densities}\;kg/m^{3}$	1780
Thickness d/μm	100
Length L/mm	2.8
Width W/mm	1.4

**Table 2:** Changes in training and validation loss of the Adam-DBNs model.

Epoch	Train Loss	Val Loss	Learning Rate
1	0.878680	0.752630	5.00 × 10^−3^
10	0.227363	0.203123	5.00 × 10^−3^
20	0.171282	0.157817	5.00 × 10^−3^
30	0.106197	0.109168	5.00 × 10^−3^
40	0.072749	0.070597	5.00 × 10^−3^
50	0.055501	0.059221	5.00 × 10^−3^
60	0.049528	0.050751	5.00 × 10^−3^
70	0.039492	0.043224	5.00 × 10^−3^
80	0.037367	0.042833	5.00 × 10^−3^
90	0.030978	0.034540	2.50 × 10^−3^
100	0.027949	0.032533	2.50 × 10^−3^

**Table 3:** Parameter specification of denoising CNN model.

CNN Layers	Kernel Size	Number of Neurons	Activation Function
**Convolutional Layer**	15 × 1	64	ReLU
**Pooling Layer**	2 × 1	-	-
**Convolutional Layer**	11 × 1	128	ReLU
**Pooling Layer**	2 × 1	-	-
**Convolutional Layer**	7 × 1	256	ReLU
**Attention**	1 × 1	256	Sigmoid
**Deconvolution Layer**	5 × 1	128	ReLU
**Deconvolution Layer**	7 × 1	64	ReLU
**Skip Connection**	3 × 1	-	-

**Table 4:** Comparison of two different method indicators under math0 samples.

Parameter	CNN	Adam-DBNs
**Spectral Flatness**	0.001395	**0.001301**
**PPMCC**	0.9872	**0.9953**
**Local Noise Energy**	0.119986	**0.020290**
**Composite Score**	69.07	**93.33**

## Data Availability

The original contributions presented in this study are included in the article; further inquiries can be directed to the corresponding authors.
